# Ready-to-use protein G-conjugated gold nanorods for biosensing and biomedical applications

**DOI:** 10.1186/s12951-017-0329-7

**Published:** 2018-01-19

**Authors:** S. Centi, F. Ratto, F. Tatini, S. Lai, R. Pini

**Affiliations:** Institute of Applied Physics, National Research Council of Italy, Via Madonna del Piano, 10, 50019 Sesto Fiorentino, Italy

**Keywords:** Gold nanorods, Antibody-antigen recognition, Protein G, Serum, Plasma, Storage

## Abstract

**Background:**

Gold nanorods (GNRs) display unique capacity to absorb and scatter near infrared light, which arises from their peculiar composition of surface plasmon resonances. For this reason, GNRs have become an innovative material of great hope in nanomedicine, in particular for imaging and therapy of cancer, as well as in photonic sensing of biological agents and toxic compounds for e.g. biomedical diagnostics, forensic analysis and environmental monitoring. As the use of GNRs is becoming more and more popular, in all these contexts, there is emerging a latent need for simple and versatile protocols for their modification with targeting units that may convey high specificity for any analyte of interest of an end-user.

**Results:**

We introduce protein G-coated GNRs as a versatile solution for the oriented immobilization of antibodies in a single step of mixing. We assess this strategy against more standard covalent binding of antibodies, in terms of biocompatibility and efficiency of molecular recognition in buffer, serum and plasma, in the context of the development of a direct immunoenzymatic assay. In both cases, we estimate an average of around 30 events of molecular recognition per particle. In addition, we disclose a convenient protocol to store these particles for months in a freezer, without any detrimental effect.

**Conclusions:**

The biocompatibility and efficiency of molecular recognition is similar in either case of GNRs that are modified with antibodies by covalent binding or oriented immobilization through protein G. However, protein G-coated GNRs are most attractive for an end-user, owing to their unique versatility and ease of bioconjugation with antibodies of her/his choice.

**Electronic supplementary material:**

The online version of this article (10.1186/s12951-017-0329-7) contains supplementary material, which is available to authorized users.

## Background

Gold nanorods (GNRs) display unique optical properties that arise from their surface plasmon resonances (SPRs), which are collective oscillations of free electrons driven at optical frequencies [1–5]. Unlike gold nanospheres, GNRs exhibit two SPR bands that reflect their anisotropic shape: plasmonic oscillations along their longer axis correspond to a so-called longitudinal SPR peak, while those along their shorter axes to a so-called transverse SPR peak. The latter typically falls at wavelengths between 510 and 530 nm and weakly depends on the size of the particles. Instead, their shapes more distinctly affect the longitudinal SPR peak, which enters the near infrared (NIR) window of greatest transparency of biological matter for aspect ratios larger than around 3.

GNRs have emerged as an innovative material of great hope in nanomedicine, in particular for imaging and therapy of cancer, because of their remarkable capacity to absorb and scatter NIR light [4–8]. In particular, their cross sections for optical absorbance exceed those of more conventional dyes, such as indocyanine green, by several orders of magnitude [9, 10]. This unique efficiency of photothermal conversion may be used to enhance the contrast in photoacoustic tomography (PAT) [5, 11, 12], for hyperthermia treatments [13–15], or to trigger a thermosensitive release in drug delivery systems [15–18]. The optical scattering of GNRs has been exploited in complementary methods of biomedical imaging, such as dark-field microscopy [19–21] and optical coherence tomography (OCT) [22, 23]. GNRs also support nonlinear optical imaging, such as two-photon luminescence (TPL) microscopy [24, 25], as well as near-field methods in combination with fluorescent or Raman tags [5, 26–30]. In addition, GNRs are well-known for their biocompatibility and convenience of conjugation with drugs and ligands [19, 31, 32]. All these features make GNRs a promising platform for applications at the crossroads of nanomedicine and biomedical optics.

Another context where GNRs have received interest is sensing of biological agents and toxic compounds for e.g. biomedical diagnostics, forensic analysis and environmental monitoring [33–37]. In particular, various examples of biosensors have been reported, where GNRs served to detect an analyte by changing colour in a suspension upon aggregation or by enhancing Raman signals [37–41].

In all cases, the availability of particles modified in such a way to hold specificity for an analyte is a key point both in biomedical optics and biosensing, especially when their use is intended in complex matrices, such as biological fluids.

The goal of this work is the presentation and critical comparison of the performances of two strategies to conjugate GNRs with antibodies for applications in biomedicine and biosensing. Both strategies rest on the modification of GNRs with a mixture of mono- and bi-functional polyethylene glycol (PEG) strands (thiolated and methoxylated or carboxylated PEG, i.e. respectively mPEG or cPEG), in order to enhance their colloidal stability, biocompatibility and readiness for conjugation with proteins by amidation. The first strategy is already well established and consists of the immobilization of antibodies on GNRs by the creation of covalent bonds between the carboxylic termini of cPEG and available amines of antibodies [19]. The second strategy is more innovative and makes use of protein G, which is grafted on cPEG for subsequent immobilization of antibodies.

The conjugation of functional particles with antibodies is a common issue. Several methods have been reported and can be broadly classified as physical, chemical or oriented immobilization [42, 43]. The physical immobilization is the most simple, but less state-of-the-art, because of its poor robustness and reproducibility. Conversely, the chemical immobilization conveys good reproducibility and coverage, but the fragment of the antibodies that is bound to the particles is rather random, i.e. the Fab fragment may be involved, thus partly hindering its availability for the antibody-antigen recognition. Different protocols have been proposed for the oriented immobilization of antibodies by preferentially binding their Fc fragment to the particles [44–46]. For instance, Puertas and coworkers [44] reported an oriented immobilization of antibodies on magnetic beads, upon oxidation of the polysaccharide domain of their Fc fragment. Parolo and coworkers [45] induced a high density of cations in the major plane of antibodies in a buffer at pH below their isoelectric point, in order to interact with anionic gold nanoparticles. Then, these ionic interactions were reinforced by the formation of peptide bonds. Both protocols proved to enhance the sensitivity of an immunoassay by as much as one order of magnitude, with respect to a random immobilization. However, the principal limitation of these protocols is their complexity, which requires qualified personnel to understand parameters as the isoelectric point of antibodies and perform a sequence of chemical reactions.

Among the possible approaches for oriented immobilization, the use of protein G or protein A is a bio-inspired alternative. These species are recombinant forms of bacterial cell wall proteins that respectively hold Fc-binding domains of staphylococcal protein A or streptococcal protein G [47–51]. The elimination of albumin and cell surface-binding domains from recombinant variants of protein G/A has established their use to separate IgG from complex samples. In particular, protein G strongly binds human, mouse, bovine, horse, monkey, porcine, rabbit and sheep IgG, more weakly interacts with rat and dog antibodies and does not bind human IgA, D, E or M and cat or chicken antibodies. Protein A weakly binds human IgA, E and M and does not bind human IgD. The use of protein G/A to immobilize antibodies may combine the simplicity of the physical methods and the advantage of the oriented approaches. Indeed protein G/A-coated GNRs may be used to bind a wide range of antibodies, through their Fc fragment, in a single step of co-incubation and without the addition of other chemicals. Alekseeva and co-workers [52] used protein A-coated GNRs for the detection of human IgG in an immunogold dot-blot assay, where the immunoglobulin was adsorbed onto a nitrocellulose membrane and protein A-coated GNRs were used for staining. More recently, Park and co-workers disclosed an inventive strategy to modify GNRs with a genetically engineered fusion protein comprising a gold-binding peptide and protein A, which replaces the cetrimonium bilayer of as-synthesized GNRs, in the presence of activated carbon [53]. Instead, some of us took advantage of the strong affinity between human IgG and protein A of staphylococcus aureus to target and impair methicillin-resistant strains of these bacteria by the combination of immunoglobulin-coated GNRs and near infrared light [54].

Here, we undertake a quantitative comparison of the efficiency of molecular recognition of antibody coated-GNRs, in the cases of a more standard chemical immobilization and an oriented approach by the interposition of protein G, at equal consumption of antibodies. In both cases, the ability of antibody-coated GNRs to bind their target was assessed by performing a direct immunoassay, by the use of an analyte labelled with alkaline phosphatase (AP), in biological fluids, such as serum and plasma. The efficiency of molecular recognition was quantified by incubation with the enzymatic substrate 4-nitrophenyl phosphate. In the presence of the analyte, AP catalyses the hydrolysis of the enzymatic substrate to 4-nitrophenol, which can be followed by spectrophotometry over time. A sheep polyclonal antibody anti-polychlorinated biphenyl (PCB)28 and the corresponding target PCB28 labelled with AP were chosen as a representative antibody-antigen model system. PCB28 is an environmental pollutant and food contaminant that has become an important target for biosensing [55, 56].

Since protein G-coated GNRs represent an attractive material for broadest deployment, we also verified their lack of cytotoxicity and compatibility with storage in a freezer, practically without any preliminary preparation. We warn the reader that limitations of protein G-coated GNRs include an inherent lack of affinity for human IgA, D, E or M and cat or chicken antibodies, a lower stability of the protein G-antibody complex [57] with respect to the more standard antibody conjugate fused through an amide bond and a need of caution in the design of sandwich immunoassays, due to a risk of exchange between capture and detection antibodies, which may be mitigated by a mindful choice of primary and secondary probes from different species. In this respect, we note that several examples of sandwich immunoassays using protein G-coated particles are available in the scientific literature [58].

## Methods

### Chemicals and immunochemicals

4-Nitrophenyl phosphate (4-NPP), diethanolamine (DEA), polyoxyethylene-sorbitanmonolaurate (Tween 20), sodium acetate, hydrogen tetrachloroaurate(III) hydrate (HAuCl_4_), hexadecyltrimethylammonium bromide (CTAB), sodium borohydride (NaBH_4_), ascorbic acid, silver nitrate, ethyl dimethylaminopropyl carbodiimide (EDC), N-hydroxysuccinimide (NHS), Tris borate-EDTA buffer, protein G recombinant, human serum and human plasma were purchased from Sigma (Milan, Italy). Acetic acid, potassium chloride and magnesium chloride were purchased from Merck (Milan, Italy). Monofunctional mPEG (HS–PEG–OCH_3_, molecular weight ~ 5000 gmol^−1^) and bifunctional cPEG (HS–PEG–COOH molecular weight ~ 5000 gmol^−1^) were purchased from Iris Biotech (Marktredwitz, Germany). A Micro BCA™ Protein Assay Kit was obtained from Thermo Scientific (Rockford, IL, USA). Sheep polyclonal antibodies against congener PCB28 and against congener PCB169 (Ab anti-PCB28 and Ab anti-PCB169) and AP-labelled PCB28 (PCB28-AP) were provided by Prof. M. Fránek, Veterinary Research Institute, Brno, Czech Republic.

All solutions were prepared by using ultrapure water from purification system UPW Refiner from Gamma 3 Ecologia (Castelverde, Italy). The composition of the buffers used for the various experiments is reported below:

- Immobilisation buffer: 100 mM sodium acetate buffer at pH 5.0 containing 0.005% (v/v) Tween 20 and 500 µM CTAB (buffer A).
- Binding buffer: 10 mM MES buffer at pH 6.0 containing 120 mM NaCl and 0.005% (v/v) Tween 20 (buffer B).
- Detection buffer for spectrophotometric measurements: 100 mM DEA buffer at pH 9.6 containing 1 mM MgCl_2_ and 100 mM KCl (buffer C).

### Synthesis and PEGylation of GNRs

GNRs were synthesized by a seed-mediated approach, as is described by Ratto and co-workers [59]. Their PEGylation was carried out by chemisorption of a mixture of mPEG and cPEG, as is reported in [19]. Briefly, after purification by two cycles of centrifugation and decantation with a dead volume ratio of ~ 1/200, GNRs were transferred at a concentration of 1.6 mM Au into buffer A containing 5 µM cPEG. This suspension was left to react at 37 °C for 30 min, then added with 45 µM mPEG and kept at 37 °C for another 90 min. After purification, GNRs were transferred at a concentration of 1.6 mM Au into buffer B.

PEG strands with molecular weight around 5000 gmol^−1^ were chosen to provide for high colloidal stability, low unspecific interactions with biological membranes and to be fit for the enhanced permeability and retention (EPR) effect, in the event of an intravenous injection for applications in oncology [19, 60]. PEGylated GNRs prove to be stable at 4 °C for over 2 weeks.

### Conjugation of PEGylated GNRs with antibodies

#### By chemical immobilization

**Fig. 1:**
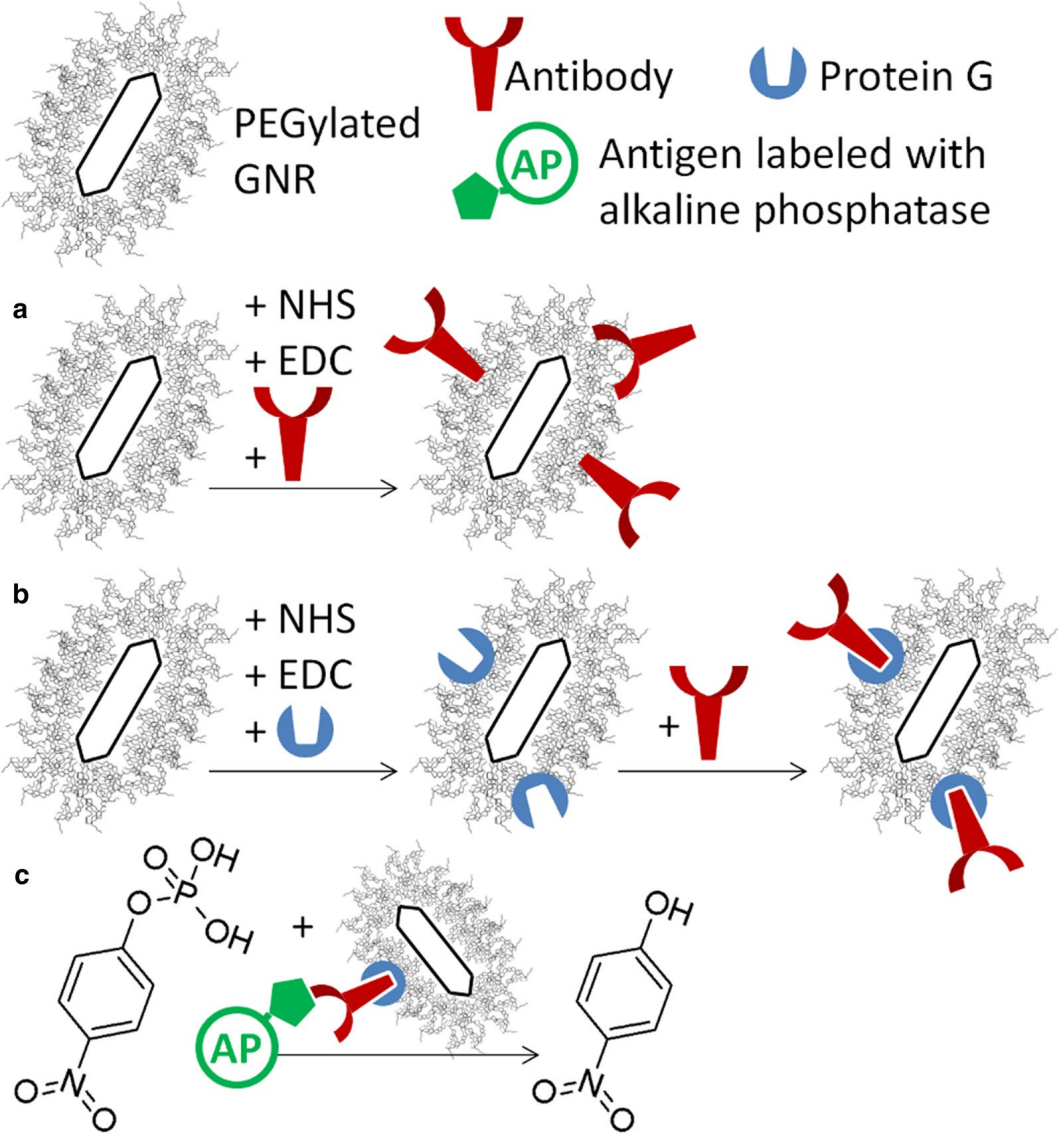
Schematic representation of covalent and oriented immobilization of antibodies onto PEGylated GNRs in **a** and **b**, respectively. The hydrolysis of the enzymatic substrate 4-NPP to 4-NP catalysed by the enzyme bound to the particles after the antibody-antigen recognition is pictured in **c**

An arbitrary volume of a suspension of GNRs at a concentration of 1.6 mM Au in buffer B was added to an equal volume of a fresh solution containing 12 mM NHS and 48 mM EDC in buffer B, which is the NHS/EDC ratio reported in [61]. After 15 min activation at 37 °C, this suspension was incubated with a double volume of a fresh solution of IgG anti-PCB28 or anti-PCB169 in buffer B. After 1 h at 37 °C, 10 mM ethanolamine was added for 30 min, in order to block any unreacted succinimide ester. After purification by two cycles of centrifugation and decantation with a dead volume ratio of ~ 1/200, particles were transferred at a concentration of 1.6 mM Au in buffer B. The supernatant from each cycle of centrifugation was directed to a quantification of the percent of unbound antibodies vs. dosed antibodies, by the use of a Micro BCA™ Protein Assay Kit [62, 63] in combination with a microplate reader (LT-4000, Labtech, Bergamo, Italy), according to the instructions from the manufacturer. A schematic representation of GNRs PEGylated and conjugated with antibodies by chemical immobilization is reported in Fig. 1a.

#### By oriented immobilization

The same protocol for the chemical immobilization of antibodies was used for the conjugation of protein G. An arbitrary volume of a suspension of GNRs at a concentration of 1.6 mM Au in buffer B was added to an equal volume of a fresh solution containing 12 mM NHS and 48 mM EDC in buffer B. After 15 min activation at 37 °C, this suspension was incubated with a double volume of a fresh solution containing 100 µg/mL protein G in buffer B for 1 h and then with 10 mM ethanolamine for 30 min. After purification by two cycles of centrifugation and decantation with a dead volume ratio of ~ 1/200, GNRs were incubated with an equal volume of IgG anti-PCB28 or anti-PCB169 in buffer B. After 1 h at 37 °C, particles were purified by two cycles of centrifugation and dispersed in buffer B. Also in this case, the supernatant from each cycle of centrifugation was directed to a quantification of the percent of unbound antibodies vs. dosed antibodies. A schematic representation of GNRs PEGylated and conjugated with antibodies by oriented immobilization is reported Fig. 1b.

The hydrodynamic size and electrokinetic potential of all particles was checked by a Zetasizer nano ZS 90 platform from Malvern Instruments (Malvern, UK).

### Affinity reaction and spectrophotometric measurements

Antibody-coated GNRs obtained by either of chemical or oriented immobilization were dispersed into 250 µL of a solution of PCB28-AP at the concentration of 3.2 mM Au, in order to form the antibody-antigen complex. After 1 h incubation at 37 °C, the suspension was washed by three cycles of centrifugation and decantation with a dead volume ratio of ~ 1/200 and then dispersed in 2 mL of a fresh solution of 1 mg/mL 4-NPP in buffer C. The formation of the enzymatic product 4-nitrophenol (4-NP) was monitored by kinetic spectrophotometry at the wavelength of 405 nm, by using a V-560 spectrophotometer from Jasco (Tokyo, Japan). A schematic representation of covalent and oriented immobilization of antibodies onto PEGylated GNRs and of the hydrolysis of the enzymatic substrate (4-NPP) by the enzyme (AP) bound to the particles through the antigen after antibody-antigen recognition is shown in Fig. 1.

### Immunoenzymatic assay in serum and plasma

250 µL serum/plasma were spiked with PCB28-AP, kept at 37 °C for 2 h and then added with particles prepared with 100 µg/mL IgG anti-PCB28 or anti-PCB169, by either of chemical or oriented immobilization, at the concentration of 3.2 mM Au. After 1 h of incubation at 37 °C, these suspensions were washed by three cycles of centrifugation and decantation with a dead volume ratio of ~ 1/200 and then dispersed in 2 mL of a fresh solution of 1 mg/mL 4-NPP in buffer C. The detection of the enzymatic product was carried out as above.

### Cell lines and culture conditions

A human cervix carcinoma line (HeLa) and a murine macrophagic line (J774a.1) were used to assess the cytotoxicity of GNRs conjugated with antibodies by either of chemical or oriented immobilization. Both lines were maintained in Dulbecco Modified Eagle Medium (DMEM) supplemented with fetal bovine serum, 100 units/mL penicillin, and 100 μg/mL streptomycin and under standard culture conditions (37 °C, 5% CO_2_, 95% air and 100% relative humidity).

### Cytotoxicity measurements

Cells were inoculated into 96-well microplates. After 24 h, their medium was replaced with fresh medium containing antibody-coated GNRs. For the assessment of cell viability, a standard 3-(4,5-dimethylthiazol-2-yl)-2,5-diphenyltetrazolium bromide (MTT) test was performed after 24 h of incubation with particles at different concentrations. Cells were incubated with 0.5 mg/mL MTT at 37 °C for 4 h and then with cell lysis buffer (20% SDS, 50% *N*,*N*-dimethylformamide, pH 4.7) at 37 °C for 3 h. The optical absorbance of blue formazan was detected at the wavelength of 590 nm by using a LT-4000 microplate reader. Cell viability was expressed as percent of MTT reduction in treated cells with respect to untreated controls. Values were displayed as mean ± SD of three different experiments, each performed in triplicate (n = 9).

### Storage of functionalized particles

Suspensions of protein G-coated or PEGylated particles at a concentration of 4 mM Au in buffer B were quenched with liquid nitrogen and then stored for 6 months at − 18 °C. After thawing in a bath at 40 °C until necessary, protein G-coated GNRs were diluted to a concentration of 800 µM Au and incubated with an equal volume of IgG anti-PCB28 or anti-PCB169 in buffer B for oriented immobilization. After 1 h incubation at 37 °C, particles were purified and used to perform the immunoassay as is described above. Instead, PEGylated GNRs were diluted to a concentration of 1.6 mM Au and, after activation and chemical binding, directed to the same immunoassay.

The kinetics of enzymatic reaction from these particles were compared to the pristine performance of their fresh counterparts as of 6 months before, in order to understand the feasibility of this approach for storage.

## Results and discussion

### PEGylation of GNRs

The PEGylation of GNRs is a key step both for biomedical and biosensing applications. PEG confers a variety of advantages, including high colloidal stability, biocompatibility, slow blood clearance after systemic injection and reactivity [64]. In addition, PEG is amphiphilic [65] and so enables suspension both in hydrophilic and lipophilic solvents.

Here, GNRs were modified with a mixture of mono- and bifunctional PEG strands (mPEG and c-PEG). We preliminarily tested several concentrations of this mixture for the PEGylation of GNRs, in terms of their efficiency to bind antibodies. After PEGylation, GNRs were conjugated with IgG anti-PCB28 (100 μg/mL) by chemical immobilization, then incubated with PCB28-AP and finally assessed by the spectrophotometric detection of the enzymatic product upon addition of the enzymatic substrate. The mixture containing cPEG and mPEG at the respective concentrations of 5 and 45 µM was chosen for all subsequent experiments owing to its high rate of enzymatic reaction and low consumption of PEG strands (see Additional file 1). Upon PEGylation with this mixture, the hydrodynamic size of as-synthesised GNRs increases by about 20 nm and their electokinetic potential reverses to a value in the order of − 10 mV, consistent with previous reports [19]. This anionic profile is ascribed to the presence of C termini from cPEG.

### Conjugation of PEGylated GNRs with antibodies and immunoenzymatic assay

#### By covalent binding

PEGylated GNRs were conjugated with IgG anti-PCB28 or anti-PCB169 by chemical binding and then incubated with PCB28-AP.

**Fig. 2:**
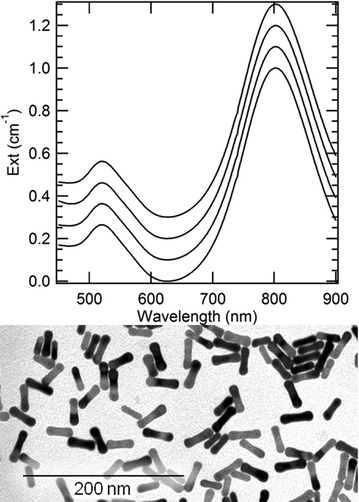
Upper panel: comparison of the spectra of optical extinction of colloidal suspensions of as-synthesised GNRs, PEGylated GNRs, antibody-coated GNRs by covalent binding and antibody-antigen-coated GNRs, from bottom to top. Spectra were offset by 0.1 cm^−1^ from each other, for the sake of comparison. Lower panel: representative TEM micrograph of as-synthesized GNRs

Particles prepared with 100 µg/mL IgG anti-PCB28 were characterized by spectrophotometry and dynamic light scattering through the various steps of the reaction. The colloidal stability and optical features of the GNRs hardly undergo any change after PEGylation, antibody conjugation and antibody-antigen recognition (see Fig. 2). We hypothesize that the use of PEG strands with MW around 5000 gmol^−1^ brings the antibodies and antigens too far away of the gold core to exert any effect on its plasmonic features. Upon antibody conjugation, the hydrodynamic size of PEGylated GNRs increases by another 20 nm, while their electrokinetic potential remains in the order of − 10 mV, consistent with prior reports [19].

**Fig. 3:**
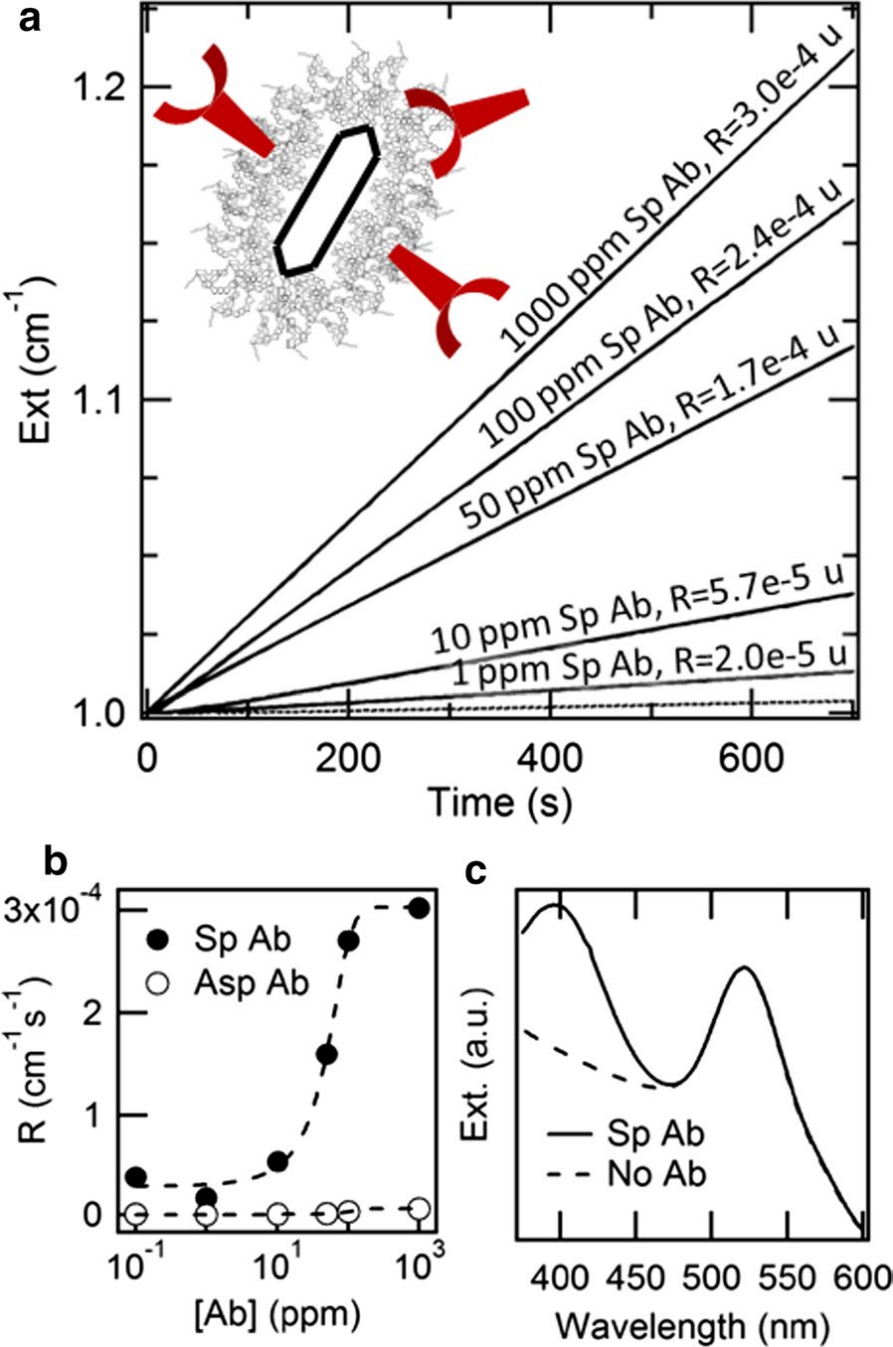
**a** Kinetics of formation of the enzymatic product for different concentrations of antibodies used for the covalent immobilization (ppm = parts per million, i.e. µg/mL in water). Rates are expressed in units of cm^−1^ s^−1^. The dotted line represents the unspecific signal for particles modified with 100 µg/mL IgG anti-PCB169 and exhibiting R = 6.2e^−6^ cm^−1^ s^−1^. The case of PEGylated GNRs without any antibody corresponds to R = 2.1e^−7^ cm^−1^ s^−1^. **b** Rate of enzymatic reaction as a function of the concentration of antibodies used for the covalent immobilization. The unspecific signal relates to particles modified with IgG anti-PCB169. **c** Optical absorbance from particles modified with 100 µg/mL IgG anti-PCB28 or anti-PCB169, in a window covering both the fingerprint of the enzymatic product around 400 nm and the transverse SPR peak of the GNRs around 520 nm

In order to quantify the efficiency of molecular recognition, we added the enzymatic substrate and assessed the formation of the enzymatic product by spectrophotometric measurements. The fingerprint of 4-nitrophenol at the wavelength of 405 nm only emerges in the presence of specific antibodies anti-PCB28, while, in the cases of unspecific antibodies anti-PCB169 or PEG alone, PCB28-AP is either hardly bound or only weakly bound and then mostly removed in the washing steps (Fig. 3c). The kinetics of formation of the enzymatic product is shown in Fig. 3a for different concentrations of antibodies used for the immobilization, together with their rates of enzymatic reaction (R). When particles are conjugated with specific antibodies anti-PCB28, the rates of enzymatic reaction are about two orders of magnitude higher than the cases of particles bound to unspecific antibodies anti-PCB169 (e.g. R = 2.4e^−4^ cm^−1^ s^−1^ vs. R = 6.2e^−6^ cm^−1^ s^−1^ when consuming 100 µg/mL antibodies) or PEG alone (R = 2.1e^−7^ cm^−1^ s^−1^). The optimization of the concentration of antibodies is documented in Fig. 3b. Different concentrations of IgG anti-PCB28 or anti-PCB169, in the range from 0.1 to 1000 µg/mL, were consumed in the immobilization. The difference between specific and unspecific binding increases with the concentration of antibodies until around 100 µg/mL, when saturation sets on. Therefore, we selected this concentration as the best condition.

In absolute terms, this concentration results into an average around 30 events of molecular recognition per particle. This figure was worked out by comparing the kinetics of the enzymatic reaction from antibody-antigen-coated GNRs with those from standard solutions of AP. In turn, the concentration of AP was calibrated from a preliminary measurement of its optical absorbance vs. that of a standard solution of bovine serum albumin, upon compensation for their relative molecular weights. Knowledge of both the number of particles [66] and molecules of enzyme per mL conveys the number of events of molecular recognition per particle, under the assumption that each event of molecular recognition brings one molecule of enzyme, as is sketched in Fig. 1.

Meanwhile, a micro BCA™ protein assay was implemented to assess the number of antibodies bound per particle, by the subtraction of unreacted antibodies during the amidation step. The fractions of antibodies left unreacted in the supernatant from the first and second cycle of centrifugation after covalent binding were (82 ± 10)% and below 1%, which amounts to an average of 40 ± 20 antibodies bound per particle, as calculated from the number of particles [66] and molecules of antibody dosed per mL. We note that this estimate is in reasonable agreement with previous reports on similar protocols for chemical binding of antibodies [62].

#### By oriented immobilization

PEGylated GNRs were conjugated with protein G, than left to interact with IgG anti-PCB28 or anti-PCB169 and finally incubated with PCB28-AP.

Also in this case, the colloidal stability and optical properties of the particles were unaffected by these reactions. The hydrodynamic size of PEGylated GNRs increases by about 10 nm upon conjugation with 100 µg/mL protein G and then by another 20 nm upon addition of 100 µg/mL IgG anti-PCB28, which maintain a slight negative electokinetic potential, in the order of − 10 mV.

**Fig. 4:**
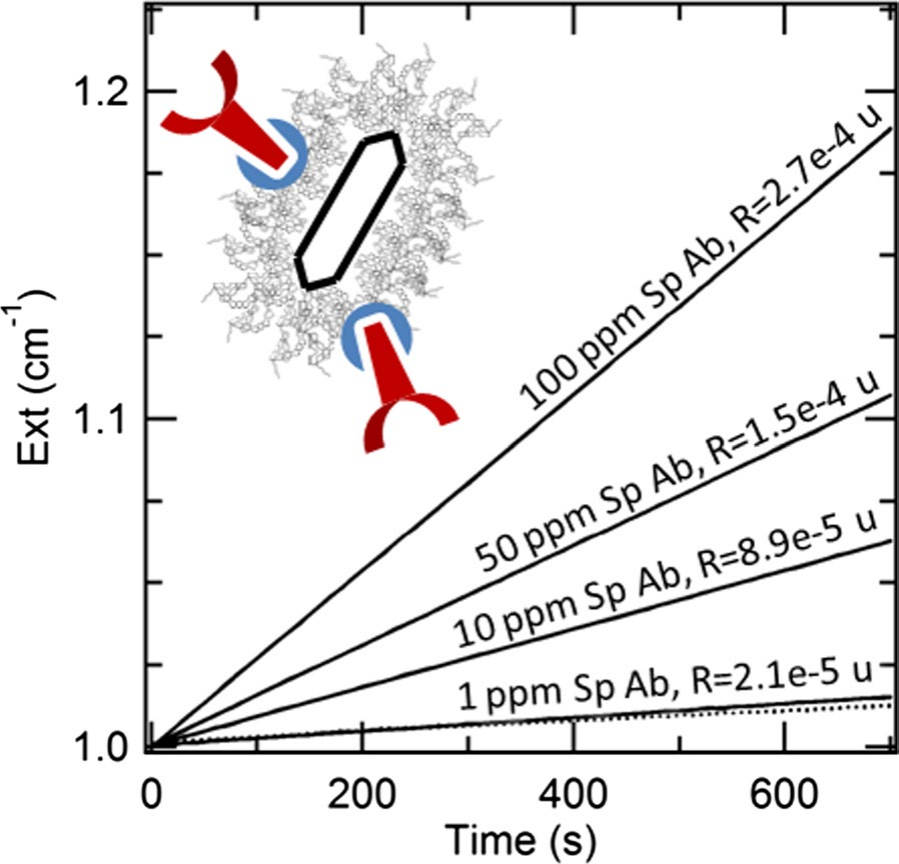
Kinetics of formation of the enzymatic product for different concentrations of antibodies used for the oriented immobilization (ppm = parts per million, i.e. µg/mL in water). Rates are expressed in units of cm^−1^ s^−1^. The dotted line represents the unspecific signal for particles modified with 100 µg/mL IgG anti-PCB169 and exhibiting R = 1.6e^−5^ cm^−1^ s^−1^. The case of protein G-coated GNRs without any antibody corresponds to R = 8.9e^−6^ cm^−1^s^−1^

The kinetics of formation of the enzymatic product is reported in Fig. 4 for different concentrations of antibodies used for the immobilization, together with their values of R. When particles are coupled to specific antibodies anti-PCB28, the rates of enzymatic reaction are more than one order of magnitude higher than the cases of particles bound to unspecific antibodies anti-PCB169 (e.g. R = 2.7e^−4^ cm^−1^ s^−1^ vs. R = 1.6e^−5^ cm^−1^ s^−1^ when consuming 100 µg/mL antibodies) or protein G alone (R = 8.9e^−6^ cm^−1^ s^−1^). Therefore, the interposition of protein G leads to an unspecific signal that is quite higher than the case of covalent binding.

In turn, the rate of enzymatic reaction for the specific combination is almost the same as for the covalent binding. Also in this case, upon incubation with 100 µg/mL IgG anti-PCB28, we estimated an average of 30 ± 20 antibodies bound per particle, corresponding to a fraction of unreacted antibodies of (86 ± 10)%, and an average around 30 events of molecular recognition per particle. We hypothesize that the advantage of an oriented immobilization may be dissipated by the random alignment and/or the steric hindrance of protein G. However, this platform remains at least as functional as the more established approach of covalent binding.

### Immunoenzymatic assay in serum and plasma

**Fig. 5:**
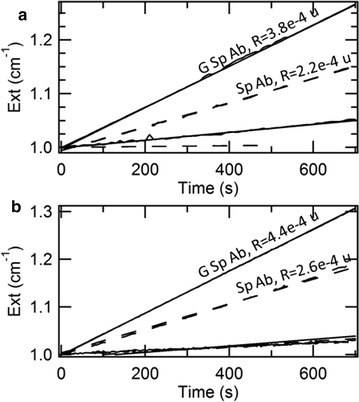
Kinetics of formation of the enzymatic product in serum (**a**) and plasma (**b**) for different protocols of immobilization, i.e. covalent (dashed lines) and oriented (solid lines) coupling to 100 µg/mL specific (upper lines) and unspecific (anti-PCB169 IgG, lower lines) antibodies. Rates are expressed in units of cm^−1^ s^−1^. In serum/plasma, the rates of enzymatic reaction are 3.8e^−4^/4.4e^−4^ cm^−1^ s^−1^ for oriented coupling to specific antibodies, 7.3e^−5^/6.9e^−5^ cm^−1^ s^−1^ for oriented coupling to unspecific antibodies, 2.2e^−4^/2.6e^−4^ cm^−1^s^−1^ for covalent coupling to specific antibodies and 8.1e^−6^/5.6e^−5^ cm^−1^s^−1^ for oriented coupling to unspecific antibodies

The kinetics of formation of the enzymatic product were also measured in complex biological fluids, such as serum and plasma. Both fluids were spiked with PCB28-AP and kept at 37 °C for 2 h, before its recognition with particles prepared with 100 µg/mL IgG anti-PCB28 or anti-PCB169, both by chemical binding and oriented immobilization. After addition of the enzymatic substrate, the detection of the enzymatic product was carried out as usual. The kinetics of the formation of the enzymatic product in serum and plasma are reported in Fig. 5. As can be seen, there is good agreement between the kinetics measured in buffer and in biological fluids. Therefore both platforms maintain their performance in real matrixes.

### Citotoxicity

**Fig. 6:**
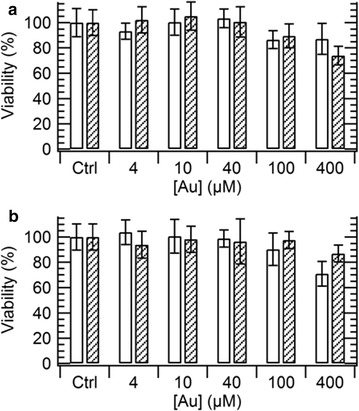
Cytotoxicity of particles modified with antibodies by covalent binding (empty bars) and oriented immobilization (shadowed bars) on HeLa (**a**) and macrophagic cells (**b**). Results are displayed as mean ± SD (n = 9)

Since GNRs are proposed for a broad variety of applications in live cells and organisms, we assessed the cytotoxicity of particles modified with antibodies both by covalent binding and oriented immobilization in a typical range of concentrations between 4 and 400 µM Au. The MTT analysis on HeLa and macrophagic cells incubated for 24 h with either type of particles is reported in Fig. 6. We found that a slight inhibition of mitochondrial activity only begins at the concentration of 400 µM Au in both cases, as is typical for PEGylated GNRs that are prepared on a lab scale, probably due to the release of contaminants [60]. Therefore, both approaches of bioconjugation are also suitable for biomedical applications. While this result is in line with previous reports for the case of covalent binding [60, 67], a green light to the use of protein G-coated GNRs in cell biology is a powerful novelty. We foresee different uses of these particles, which may be distributed for easy bioconjugation with antibodies of choice of an end-user or even as secondary labels.

### Storage of functionalized particles

**Fig. 7:**
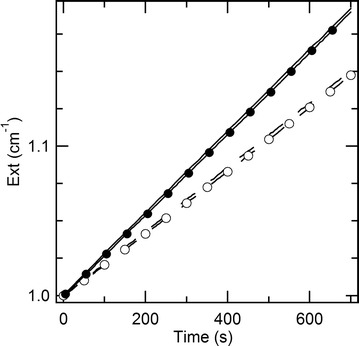
Kinetics of formation of the enzymatic product for fresh (lines) and freeze-thawed (lines and sparse symbols) particles after 6 months at − 18 °C modified with antibodies by covalent binding (dashed lines and empty symbols) and oriented immobilization (solid lines and full symbols)

The perspective to deploy these particles as a versatile tool also depends on the feasibility of their storage. We compared the performances of antibody-coated particles both by covalent binding and oriented immobilization before and after 6 months in a freezer. After freeze–thawing, the onset of flocculation was ruled out by dynamic light scattering that did not detect any variation in terms of hydrodynamic size (within 5 nm). Then, the ability to recognize PCB28-AP was assessed as usual. Figure 7 displays the comparison between the kinetics of formation of the enzymatic product of as-prepared and freeze-thawed particles modified with antibodies both by covalent binding and oriented immobilization. The rates of enzymatic reaction are almost identical after at least as many as 6 months. Therefore, this method for storage is conservative for the functional features of antibody-coated particles. Different authors have disclosed the notion to store GNRs modified with functional coatings, such as PEG [68] or quaternary amines [69], by freeze drying. Freeze drying is probably more convenient than freezing to the extent that the specimens may be stored at room temperature. However, here we tested freezing, because our protein G and antibodies were recommended for storage in an aqueous environment at − 18 °C. To the best of our knowledge, freezing is a new possibility to store antibody-coated GNRs. We warn the reader that the freeze-thaw cycle should be as rapid as possible, in order to prevent the onset of flocculation.

## Conclusions

Antibody-coated particles are widely used both in biomedical and biosensing applications. We have quantified and compared the performances of GNRs modified with antibodies by covalent binding and oriented immobilization by the interposition of protein G, in terms of their efficiency of molecular recognition and extent of unspecific signal, in the context of the development of a direct immunoenzymatic assay. In the case of covalent binding, the difference between the rates of enzymatic reaction for specific and unspecific interactions is about two orders of magnitude and decreases when moving from buffer to serum and plasma. Instead, this difference is only about an order of magnitude when protein G is interposed, but does not change much from matrix to matrix, so that, in plasma, the oriented immobilization is even more specific than its more standard covalent counterpart. We have estimated that both platforms enable an average around 30 events of molecular recognition per particle both in buffer and biological fluids. The principal merit of protein G-coated GNRs is their versatility and ease of use by unskilled personnel, since the antibody immobilization only involves one step of mixing without the addition of other chemicals, as is the case for less robust physical methods. Their main limitation is a lack of affinity for human IgA, D, E or M as well as IgG from relevant species as cat or chicken. In addition, we have found that GNRs modified with antibodies by oriented immobilization are as nontoxic as PEGylated GNRs and protein G-coated GNRs may be stored for months in a freezer without any effect on their functional features. Taken together, our results demonstrate that the modification of GNRs with protein G may become a translational solution for their broadest deployment for a variety of applications in nanomedicine and biosensing.

## Additional file

**Additional file 1.** Additional details on the PEGylation of the gold nanorods.
