# Supplementary material for: Ready-to-use protein G-conjugated gold nanorods for biosensing and biomedical applications
Source: J Nanobiotechnology. 2018 Jan 19;16:5. doi: 10.1186/s12951-017-0329-7 (PMC5775603; doi:10.1186/s12951-017-0329-7)
Supplement: Supplementary file 1 — Additional file 1. Additional details on the PEGylation of the gold nanorods. [file 12951_2017_329_MOESM1_ESM.pdf]

Additional file 1

Figure A1 displays the rate of enzymatic reaction for gold nanorods that were PEGylated with 1.6 mM Au and 5 / 45, 50 / 450 or 500 / 4500 µM cPEG / mPEG, then covalently coupled to 100 µg/mL anti-PCB28 antibodies, exposed to PCB28-alkaline phosphatase and finally suspended in its enzymatic substrate, with the intent to rationalize the consumption of heterobifunctional PEG strands. The definition of all acronyms and more experimental details are given in the main text.

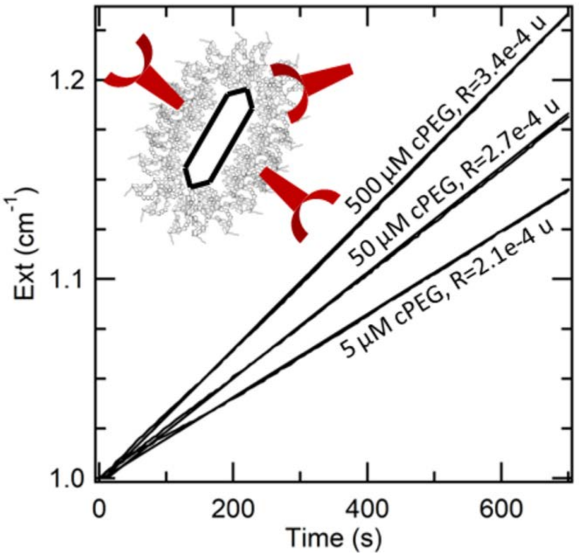

Figure A1: Kinetics of formation of the enzymatic product for different concentrations of cPEG and mPEG consumed in the PEGylation ([mPEG] = 9*[cPEG]). Rates are expressed in units of cm^{-1}s^{-1}.

We note that a decrease in the consumption of heterobifunctional PEG strands and relevant costs by as much as 100 folds leads to a decrease in the efficiency of molecular recognition of the final construct by as little as 40%. In turn, at current market prices, a reaction involving 1.6 mM Au gold nanorods, 5 µM cPEG and 45 µM mPEG costs around 170 €/L in $HAuCl_4$ (Sigma, Milan, Italy), 17 €/L in cPEG (Iris Biotech, Marktredwitz, Germany) and 64 €/L in mPEG (Iris Biotech, Marktredwitz, Germany), which makes the PEGylation sustainable, when benchmarked against the overall value of consumables. For these reasons, we believe that these conditions are an excellent trade-off between performance and costs.

Figure A2 shows a picture of a 0.2% agarose gel containing gold nanorods PEGylated with different mixtures of 50 µM polymer strands and biased with a potential of 130 V. In Tris borate-EDTA buffer solution at pH 8.3, the electrophoretic mobility of PEGylated gold nanorods undergoes reversal as the fraction of cPEG increases and provides an anionic profile, which suggests the possibility to tailor the density of carboxylic functions to different applications that may benefit from more stealth profile from mPEG or more anchoring sites from cPEG.

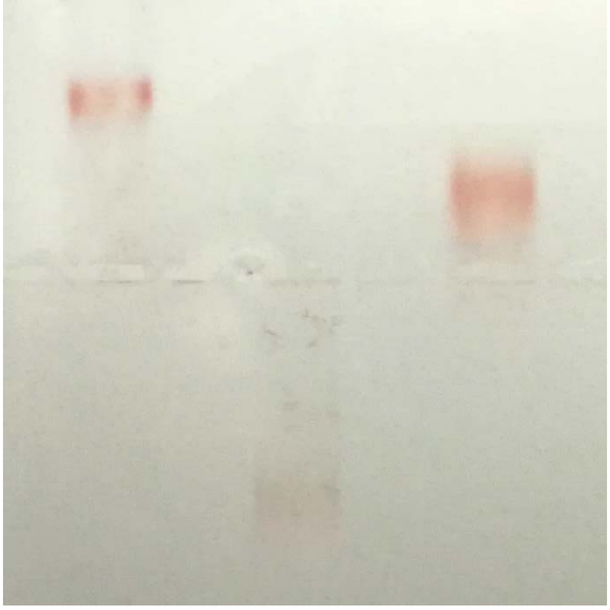

Figure A2: Picture of an electrophoretic chamber featuring gold nanorods PEGylated with 50 µM cPEG; 50 µM mPEG, or 25 µM cPEG and 25 µM mPEG, from left to right. The positive pole is upwards and the negative pole is downwards.
